# Editorial: Exploring the Potential of Particle Radiotherapy: Helium, Neutrons, Carbon, and Other Heavy Ions

**DOI:** 10.3389/fonc.2021.740974

**Published:** 2021-07-29

**Authors:** Timothy D. Malouff, Stephanie E. Combs, Daniel M. Trifiletti

**Affiliations:** ^1^Department of Radiation Oncology, Mayo Clinic Florida, Jacksonville, FL, United States; ^2^Department of Radiation Oncology, Technical University of Munich, Munich, Germany

**Keywords:** particle, carbon, helium, neutron, heavy ions, radiation

## Overview

Radiation therapy is a cornerstone modality in the treatment of malignant diseases. Since the inception of the field over a century ago, clinicians and researchers have focused on improving the therapeutic ratio of radiation therapy, therefore minimizing toxicities while maximizing tumor control. The use of particle therapy to improve the therapeutic ratio can be traced to 1946, when Dr. Robert Wilson first proposed the use of accelerated protons and heavy ions for oncological treatments in a landmark paper (1). Today, clinicians and researchers are investigating the use of a variety of different ions for therapeutic use, including protons, carbon ions, fast neutrons, boron neutron capture, and multi-ion therapy (2). Each of the heavy ions have unique radiobiological and physical properties that must be taken into consideration, although they all share some common features, such as a high linear energy transfer (LET) and relative biological effectiveness (RBE). These properties theoretically make heavy ions more potent at causing DNA damage and hopefully improving tumor control (3, 4)

Our topic accepted a total of 17 articles from 48 authors, demonstrating the emergence and importance of particle therapy in providing the best care for patients. Our topic can be divided into the following topics:

## Proton and Carbon Ions

Much of our clinical experience with particle therapy involves proton therapy, which is widely used in the US and throughout the world, followed by carbon ion radiotherapy, with centers treating throughout Europe, Asia, and a planned center in the US (5–9). Carbon ion radiotherapy, which is the focus of our Research Topic, takes advantage of the Bragg peak, a sharp lateral penumbra, high LET, and high RBE to maximize cell kill while minimizing normal tissue irradiated (3). Clinical studies have suggested safety and efficacy of carbon ions in the treatment of a variety of malignancies (2).

Our topic includes three excellent clinical reviews describing the clinical experience of particle therapy for skull base sarcomas (Yang et al.), adenoid cystic carcinoma of the nasal cavity and sinuses (Hu et al.), and meningiomas (Li et al.). Additionally, studies by Huang et al. and Yang et al. demonstrate molecular mechanisms for the bystander effect and abscopal effect, an area of excitement and a potential niche for heavy ion therapies. Furthermore, Sun et al. and Toppi et al. report on methods to evaluate dose distribution following carbon ion radiotherapy.

## Fast Neutron Therapy

Although not commonly used, fast neutron therapy is another area of interest, as neutrons have a high have a high LET and RBE despite not exhibiting a Bragg peak (10). Jones authored a comprehensive review on the clinical radiobiology of fast neutron therapy, as well as the historical and future concerns of implementing neutron therapy.

## Boron Neutron Capture Therapy

Although first proposed in 1936, boron neutron capture therapy (BNCT) has experienced a resurgence in interest (11). BNCT is based on the principle of irradiating nonradioactive boron-10 with neutrons, leading to the production of a lithium-7 and an alpha particle. The alpha particle is a form of high LET radiation that deposits energy over the distance of about the diameter of one cell, therefore selectively targeting tumor cells while avoiding normal tissue toxicity. This technique has largely been limited due to the limited selectiveness of the boron compounds (11–13). The review by Malouff et al. describes the clinical experience and future directions of BNCT.

## Multi-Ion Therapy

Although much of our clinical experience is based on individual particles used alone, there is a resurgence of interest in combining the use of multiple ions to take advantage of the unique characteristics of each ion. For instance, helium, neon, silicon, nitrogen, and argon were all studied at the Lawrence Berkeley National Laboratory in the 1970s (14). In the review by Ebner et al., the authors describe the initial work, as well as the challenges associated, with combining these ions in a single treatment to best distribute high LET regions in tumors while minimizing high LET regions in normal tissues or areas of subclinical disease.

## Conclusion

Overall, particle therapy represents an area of excitement in radiation oncology, as is evidenced by the excellent articles listed above. We hope that our Research Topic promotes discussion, identifies gaps in knowledge, and inspires future generations to continue investigating the therapeutic use of heavy ions.

## Author Contributions

All authors contributed to the article and approved the submitted version.

## Conflict of Interest

The authors declare that the research was conducted in the absence of any commercial or financial relationships that could be construed as a potential conflict of interest.

## Publisher’s Note

All claims expressed in this article are solely those of the authors and do not necessarily represent those of their affiliated organizations, or those of the publisher, the editors and the reviewers. Any product that may be evaluated in this article, or claim that may be made by its manufacturer, is not guaranteed or endorsed by the publisher.
